# The Future of Dentistry

**Published:** 1904-12

**Authors:** M. R. Harned

**Affiliations:** Rockford Ill.


### THE FUTURE OF DENTISTRY.

M. R. Harned, D. D. S., Rockford Ill.

A treatise upon such a subject as the one chosen by the essayist, must necessarily be speculative; dealing in futures is always somewhat hazardous and especially where there are so many elements to modify the complex question of personality with which we have to deal.

My hope is to follow along lines natural and fundamental hoping our topsy-turvy civilization with its complexities will not entirely divert us from the course, if the course suggested be really best as well as ideal.

E. H. Griggs in his "The New Humanism" says, "An ideal is the most practical thing in the world, for it is a force behind action that must be reckoned with by the frankest materialist." The ideal which actuates the paper is not making of money, making a perfect artificial denture, or the ideal filling, or making a living, but rendering service, and making of ourselves men.

Progress is made only by assuming something and striving for it, leaving the old and established, the certainty, behind and striving for the unknown, the ideal.

This choice or ability to choose, is one of the striking distinguishing traits between man and the lower orders of animals. This ability to have an ideal is what Charles Ferguson calls the "affirmative quality of the intellect." This is the quality which begets aspiration, gives vigor and hope and realizes progress. This quality is offset by the passive quality, which is satisfied with letting "well enough alone," with conserving what we have it is purely animal:. For illustration, the horse is satisfied with the same kind of food as the horse of a thousand years ago: The fish is still satisfied with the environment of ages and deposits its spawn where the greatest percentage of young is devoured before fairly started in life: The bird builds its nest after the fashion of its parent, the construction has not materially changed in the knowledge of man: The mouse still steals about our pantry even though all his predecessors have died for the trick: The gopher still burrows in the ground, though boys and dogs dig or drown them out: In other words they follow the line of heredity that negative side of life, which constantly tends to conserve, or drag back to the original type.

Not so with man who is endowed with that God given quality of "Affirmation." He is never satisfied with his diet, his environment, the habits of the family, the housing and care of self and family is always luring him on; he wants his children to have a better chance in life than he had, he wants to constantly reduce the hazard for them; he wants them to be greater and better than he. And woe unto him if he does not, for this feeling of unrest and aspiration is the very key which unlocks natures store-house of food and fuel and minerals and develops arts and sciences and mechanics and reveals to him the phenomena of the potentialities of nature and of life: It discovers to him the law of God's sublime "Drama of Life" which is "development."

That dentistry as an art is an old one, has been demonstrated by recent excavations of Etruscan tombs, how old would be difficult to say but several thousand years at least.

A wag has recently said that "The first historical record of dentistry which could be considered authentic was in the times of the tyrant Nero, who, becoming satiated with seeing christians beheaded and burned at the stake and torn to pieces by wild animals, when more victims were brought, gave orders that they be dragged to a dentist and their teeth extracted, the pain was so excruciating, so much worse than death, that the tyrant was never known to have another victim slain."

Thus we see that dentistry began saving lives a long time ago.

Art broadly defined is any service which contributes to the happiness or welfare of mankind. "All other service (in the language of a philosopher) is crime."

The dental art, with all art, has its origin in human need.

Back in the primitive life when all life was religious expression, and largely propitiating the wrath of gods, it is not at all improbable that when teeth ached, libations were poured, incantations sung, and sacrificial offerings made. May-hap some old woman (who was not very useful) became crazed with tooth-ache and was looked upon as bewitched, hence was offered as a sacrifice on some funeral pyre or altar to propitiate the wrath of God. This sort of performance might have gone on and probably did until some skeptical fellow, who had a tooth-ache, got it knocked out in a fight, thus learning that tooth-ache was not God's wrath, though the relief might be the other fellow's wrath.

The priesthood recognizing the danger to their business privileges, denounced the fellow as a heretic, but in spite of the denunciation the truth had come to light, and while the struggle against enlightenment continued long and fierce, other experiences of other people, together with the protest of a few skeptics who did not believe that prayers would cure tooth-ache, finally pushed superstition back, and the priests or medicine-men from denouncing the growing custom of getting of their healing art the extraction of teeth.

The foregoing is practically an epitome of the great struggle which has gone on in advancing civilization. Every step in progress and enlightenment has been fought by the priesthood in the name of religion, (as you will find by reading Andrew Dixon White's "Conflict between Religion and Science") but in reality because it would interfere with their privileges of saying prayers and getting paid for it, which had nothing to do with religion.

But in spite of this opposition, men with affirmative intellects have fought the sin of ignorance, have learned the laws of pain and developed the art of rendering service and alleviating suffering.

And for rapid strides dentistry has taken the lead. We have seen the evolution of extracting teeth on any occasion of pain, with a stone and stick, through the turn key and elevator periods to the modern forcep, and from the extraction of all teeth that pain to the extraction of deciduous, supernumerary and loose teeth. The extraction of other than these is becoming more and more rare, and in most cases today is due to nothing except the financial consideration; we can successfully treat and save practically anything in shape of a tooth that is solid in the jaw.

What a parody on our civilization which does not permit the preservation of all one's parts and faculties, providing he is willing to work, simply because of the unjust distribution of products.

We recognize that not more than half the dental work is now being done that ought to be done and it would require a much larger force of dentists than we now have to meet this demand, but these would be forthcoming if the conditions were right.

We have seen the evolution of artificial dentures from the carved ivory imitations, through the metal dentures with porcelain soldered on, the vulcanite, and celluloid plates with porcelain teeth, to the modern continuous gum case, which is the perfection of artificial substitution, but artificial just the same, and we look forward to the time when there will be none of them.

We have seen the development of tooth treating and saving, from camphor and liniment and creosote, and burning with a heated knitting needle, to modern methods of palliation intelligently applied, with knowledge that all tooth aches are not alike; the modern method of pulp extripation and root treating and filling, only twenty years ago was looked upon by many dentists as very doubtful treatment, reasoning that a tooth from which the pulp was gone was a foreign substance, and would soon be exfoliated. We have seen the filling of teeth pass from fitting a lead bullet into a cavity to gold rolled into soft cylinders and wedged in; the use of gutta percha, Royal Succeedanium, (or amalgam); we have seen this compounded of every known metal that was cheap, including copper amalgam, and used in any and all teeth and cavities; we have seen it condemned because so abused and finally brought up to its present standard of excellence, which makes it a good tooth saver if handled with skill and used with discretion. We have seen the many preparations of cement including oxychloride, and oxyphorphates, and we still hope for something better in this line. We have seen the development of the use of cohesive gold which enables us to restore contours so perfectly. The systematic and scientific development of cavity preparation, which not only contemplates removal of decayed portion of tooth and replacement with artificial substitute, but considers withstanding various strains and stresses, and the extension of filling for prevention of future decay. And through the development of electric appliances has come the use of porcelain, that most artistic of artificial substitutes for missing tooth structure; it has its limitations, but its artistic effects, its insulating quality, the comfort of cavity preparation, its quality of sustaining frail tooth structure by aid of cement, renders it, to date, preeminently the artistic filling. Our great need at present for this kind of work is a cement without color.

We are and have been passing through a mechanical and scientific era, which has affected all lines and especially dentistry. What could be more wonderful than the mechanical development in dentistry in the past twenty-five years, from the old boiler to the modern vulcanizer with its automatic regulator, from the old set of three chisels to Black's set of 42? From the old spittoon which was practically impossible to clean, to the modern fountain cuspidor with its equipment, from the old barber chair to the modern operating chair and from the burr operated by hand or with cross-bow to the modern electric engine, are strides almost inconceivable in this short time. Mechanically we have arrived at the place where to want a thing means you can have it if you will pay for it.

The growth has been astounding, the development marvelous.

By dint of hard work, patient toil and sacrifice on the part of our predecessors, and colaborers, dentistry has been brought from the mere trade of an itinerary to the standard of the highest profession; it has been brought from a tooth destroying executioner to a beneficent, palliating, tooth-saving, health-saving, life-saving profession.

But we are not satisfied and are not going to be satisfied, and we propose not only to uplift our profession, and the "personnel" thereof but we intend to uplift humanity.

Among the things we desire and lend our influence to help along is our public school system; we desire to see it brought more and more into the line of the Froebel idea, wherein the child is taught to express rather than being impressed, so that when he takes up a life work he will know that he has adaptability. Then when he enters dentistry it will not be because he doesn't know what else to do, but because he can thereby express himself and help humanity.

Then we hope to see our college training which is now so far in advance of training for other professions, conducted by national universities, which will not have to consider expense and will procure the services of the best teachers, giving them an opportunity to progress even if it doesn't pay in dollars.

We also hope to see the curriculum prescribed, one which shall be the highest and best, which the knowledge of the day will suggest, and hope that the student will be given his degree as soon as he has done the prescribed work, thus giving him the benefit of application and conscientious work or special adaptability, on the other hand it will punish by detention him who wastes his time, while our present system punishes the good student instead.

Then we hope to see some other method of rewarding a man for original ideas than the present system of patents, which if he is able financially to protect simply permits him to tax the dentists and indirectly the people. Why not have a system of rewards, or medals, a roll of honor, a fellowship in the "Society of Affirmative Intellects." Any of these would be preferable to the doubtful realization of money after getting through courts, unless the man's ideal is "dollars," if so he should have no place in a profession. As a matter of fact there is compensation enough in the realization of good done to humanity.

One of the things which we as leaders in progress must strive for is a reorganization of society so that a few individuals will not grow rich at the expense of the many, so that each individual must work for what he has and no one or few will be allowed to monopolize nature's resources, they should belong to all and in justice do. Some of us have been laboring under the delusion that we can pass the crowd and scale the wall and win the goal, but the modern science of sociology has discovered that this cannot be done, the enlightenment must "leaven the whole lump," or all will fall together.

And this law, with regard to exclusion, applies within the profession the same as outside and it is my honest opinion that if the right hand of fellowship were extended to the young men entering the profession by the older practitioners and the societies, fewer of them would ever start upon an unprofessional career; most of them start because they feel it is necessary to get a living and would be very glad to give it up. We need more association and less exclusion. When we are in competition we need to see each other often to keep out the idea that the other fellow is knifing us, that he is unfair.

One of the things which as a profession we hope soon to inaugurate in a much larger degree is the proper education of children and parents as well, on the care of the teeth. It will of course take generations for it to become a habit, as it now is to wash before eating and sleeping, but the repeated and constant dinging away at these things has its influence. Care of teeth should be incorporated in our public school system, as gymnastics and other healthful exercises are.

As less and less teeth are lost we will find our profession becoming simpler, we will devote our time to the analysis of the fluids of the mouth of our patients, to the effects of various food substances, the effect of habits, as smoking, chewing tobacco, gum or aught else, the effect of variation of temperature, if any, the effect of moods, of laughter and tears, of love and hate, of hope and discouragement, in fact the effect of thought. We will strive to know the effects of all the environments and potentials of life upon the teeth, in relation to conservation. Our work will be preventive rather than reparative.

We will study the causes of disease uncertainly must get back of simply curing a disease, we must prevent, and then who knows but we may conduct a tooth insurtil we will even know the cause of pyorshea alveolaris, of neuralgia, carcinoma, etc., and study remedial agencies until we know how to prevent these troubles. We ance establishment for our patients, even now this is an important part of our business.

If we would make the future of dentistry what its present impetus and potentiality would imply, each must resolve that he will pay the price by sacrificing something of comfort and pleasure to the common cause of the profession and humanity. We must not rest upon the laurels won, and try only to conserve what we have gained for that is death. We must not be simply the passive foam upon the beach, inflated with a little false pride, but be a wave on the bosom of the great sea, a part of the great universal active whole.

